# Phytochemical profiling of *Rosmarinus officinalis* aerial parts and exploring its *in vitro* wound healing activity and network pharmacology

**DOI:** 10.1038/s41598-025-31482-4

**Published:** 2026-01-05

**Authors:** Shaza H. Aly, Aya A. Mohamed, Mariam Ehab, Alaa M. AbdElaziz, Nouran Ehab, Eman Salem, Manar Amgad, Omnia Mohamed Gaber, Hager Amer, Sara Saeed Kotb

**Affiliations:** 1 Department of Pharmacognosy, Faculty of Pharmacy, Badr University in Cairo (BUC), Cairo, 11829 Egypt; 2 Faculty of Pharmacy, Badr University in Cairo, Cairo, 11829 Egypt

**Keywords:** Antioxidant, HPLC/MS, Rosmarinus officinalis, Wound healing, Anti-inflammatory, rosmarinic acid, Network pharmacology, Biological techniques, Plant sciences

## Abstract

The present study thoroughly assessed the wound healing efficacy of fractions derived from *Rosmarinus officinalis* through phytochemical profiling, antioxidant assays, and *in vitro* scratch wound models, along with network pharmacology to identify target genes. HPLC/MS analysis identified rosmarinic acid as the predominant phenolic compound, alongside diterpenoids (carnosic acid, carnosol) and flavonoids (cirsimaritin, diosmetin). The total extract exhibited the highest total phenolic content (106.56 µg gallic acid eq/mg), while the ethyl acetate fraction (ROE) contained the highest flavonoids (43.7 µg rutin eq/mg). Antioxidant assays revealed fraction-dependent efficacy: the *n*-butanol fraction (RON) showed superior (Ferric Reducing Antioxidant Power) FRAP activity (637.727 µM TE/mg), whereas ROE demonstrated potent radical scavenging (DPPH (2,2-diphenyl-1-picrylhydrazyl) IC₅₀: 22.81 *µg/*mL; ABTS (2,2’-azino-bis(3-ethylbenzothiazoline-6-sulfonic acid) IC₅₀: 33.6 *µg/*mL). *In vitro* scratch assays on human skin fibroblasts (HSF) highlighted ROE and RON as the most effective fractions, reducing wound widths to 0.42 ± 0.04 mm and 0.41 ± 0.005 mm, respectively, within 24 h at 10 *µ*g/mL. These fractions also suppressed LPS(Lipopolysaccharide)-induced nitric oxide production in macrophages by > 70%, underscoring anti-inflammatory synergies. Furthermore, utilising network pharmacology, we identified ten hub target genes associated with wound healing, including IL6 and 1B (Interleukin‑6, -1B), TNF (Tumor Necrosis Factor) and FN1(Fibronectin 1). The findings establish that solvent polarity critically influences bioactive compound recovery, with semi-polar fractions (ROE, RON) optimally balancing antioxidant, anti-inflammatory, and fibroblast-migratory properties for wound healing applications. As a conclusion, *R. officinalis* is a great natural candidate for valuable bioactive components with promising anti-inflammatory, wound healing, and antioxidant properties. Further phytochemical studies should be performed to isolate the responsible compounds and investigate their mechanism of action.

## Introduction

The medicinal potential of plant bioactive compounds is evidenced by their roles as antioxidants, anti-inflammatory agents, antimicrobials, and modulators of metabolic and signaling pathways^1,2^. Innovation in drug development, nutraceuticals, and functional foods is supported by the continuous investigation of plant biodiversity, which continues to produce new bioactive compounds with distinctive structures and functions^3,4^. Research on plant phytochemicals is still ongoing to find potential herbal remedies that can treat a wide range of many ailments^5,6^.

*Rosmarinus officinalis* L., generally referred to as rosemary, is a highly regarded aromatic and medicinal herb prevalent in the Mediterranean region; it is a member of the Lamiaceae family^7,8^. Historically, rosemary has been recognised since ancient times and was even mentioned in Egyptian, Greek, and Latin writings^7^. Its name is believed to derive from the Latin words “ros” (dew) and “marinus” (sea), translating to “dew of the sea,” likely due to its presence on calcareous soils in warm coastal regions^7^. *R. officinalis* contains a variety of bioactive compounds, including 1–2% essential oil, flavonoids such as cirsimarin and cirsimaritin, approximately 8% tannin, diterpenoids, triterpenoids and polyphenolic acids^9,10^. Notable polyphenolic acids identified comprise rosmarinic acid, caffeic acid, chlorogenic acid, ferulic acid, and protocatechuic acid. Rosmarinic acid, a principal constituent, is composed of a dimer of caffeic acid conjugated with hydroxycaffeic acid^10,11^. Notable diterpenoids, referred to as “bitter principles,” include abietane-type compounds such as rosmanol, carnosic acid, and carnosol^7^.

Regarding the major compounds identified in *R. officinalis*, they showed promising wound healing properties. Where, topical rosmarinic acid may be useful in promoting wound size reduction and limiting scar formation^12^. Another study demonstrated that rosmarinic acid-loaded chitosan-graphene nanoparticles accelerated wound healing by combining antimicrobial effects and improved tissue repair mechanisms, surpassing conventional rosmarinic acid or gel treatments alone. This innovative nanosystem could offer a potent tool for chronic wound management^13^. Carnosol and carnosic acid exhibit protective effects in various inflammatory disease models, including wound healing, neuroinflammation, arthritis, and metabolic disorders, by targeting critical signaling mechanisms and oxidative stress pathways^14^. Furthermore, the presence of flavonoids in *R. officinalis* constitutes a significant component, as these promising natural substances facilitate wound healing through several biochemical and cellular pathways, including anti-inflammatory, antioxidant, and antibacterial characteristics^15^.

A variety of studies have examined the pharmacological effects of *R. officinalis* extracts, revealing a wide range of biological properties^16^. These include antioxidant, anti-inflammatory, antidepressant, antibacterial, antifungal, antiviral, and antiallergic properties^17–19^. Furthermore, *R. officinalis* has shown neuroprotective, hepatoprotective, nephroprotective, antiproliferative, antitumor, immunomodulatory, antihypertensive, anti-ischemic, hypolipidemic, hypocholesterolemic, hypoglycemic, antifibrotic, and radioprotective effects^20^. In ethnopharmacology, traditional applications encompass memory enhancement, alleviation of rheumatic pain, treatment of headaches, stomach discomfort, dysmenorrhea, epilepsy, cognitive disorders, and hysteria^19^.

*R. officinalis* holds significant potential as a wound-healing agent^21^. Its efficacy has been evaluated in various wound models, including infected, excisional, and incisional wounds, as well as pressure ulcers and burns. *R. officinalis* essential oil and extracts contribute to accelerated wound healing through multiple mechanisms, including antibacterial, anti-inflammatory, and collagen-promoting properties^8^.

Elucidating the mechanisms by which natural products alleviate diseases is highly complex, due to their intricate metabolome, potential metabolite synergism, and multi-target effects^22^. Network pharmacology analysis effectively addresses this challenge by enabling the construction of compound-target gene-disease networks. This facilitates the identification of multiple targets and pathways involved in the metabolites’ action^23,24^.

Antibacterial action controls infection, anti-inflammatory action resolves destructive inflammation, and collagen-promoting action rebuilds the tissue – each step sequentially enabling the next for efficient wound closure and strength restoration.

The antibacterial, anti-inflammatory, and collagen-promoting properties function synergistically to enhance wound healing efficiency^25^. The antibacterial effect inhibits infection, eliminating an important barrier that initiates harmful inflammation and tissue damage^26^. Anti-inflammatory properties subsequently mitigate excessive inflammation, establishing a steady environment essential for the subsequent phase^27^. Ultimately, collagen-stimulating activity promotes the essential reconstruction of tissue structures and function^28^. Each attribute sequentially enabling the next for efficient wound closure and strength restoration.

**Fig. 1:**
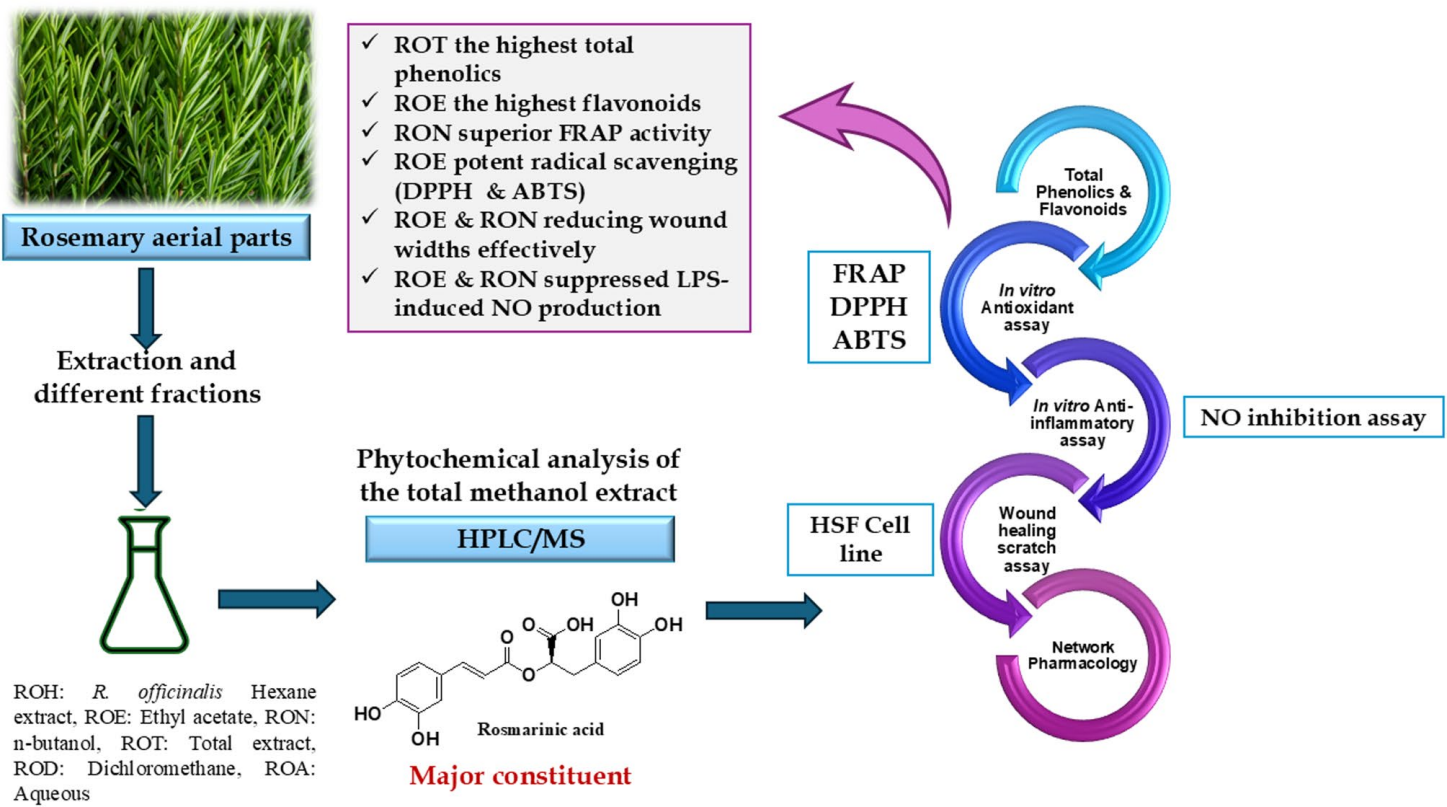
A schematic diagram representing the summary of the study on *R. officinalis* aerial parts.

The objective of this research was to comprehensively elucidate and compare the *in vitro* wound healing abilities of different fractions of *R. officinalis* aerial parts, including their antioxidant and anti-inflammatory effects. Along with unveiling the compositional profile of the total extract of *R. officinalis*, network pharmacology analysis was used to confirm and elucidate the mechanistic background in wound healing of *R. officinalis* extract (Fig. 1).

## Results and discussion

### HPLC/MS analysis of the *R. officinalis* total extract

The LC-MS/MS analysis of *R. officinalis* total methanol extract revealed a diverse profile of bioactive compounds, with phenolic acids, diterpenoids, and triterpenoids being the predominant classes identified. The HPLC-MS/MS analysis of the *R. officinalis* extract yielded structural information for the fifteen peaks with the highest percentage area, determined based on previously reported data. Rosmarinic acid (9) (*m/z* 359.24/361.21) was detected as key phenolic acid in both negative and positive ionization modes, consistent with its reported presence in *R. officinalis* extract^10^. Notably, diterpenoids such as carnosol quinone (2) (*m/z* 327.26), Carnosol (3) (*m/z* 329.28), rosmanol (6) (*m/z* 345.24), and its isomer epirosmanol (12) (*m/z* 345.26) were identified, highlighting structural diversity within this class. These compounds, alongside methyl carnosate (7) (*m/z* 345.20), ursolic acid (14) (*m/z* 455.43) and betulinic acid (15) (*m/z* 455.40), which are recognized for their antioxidant and anti-inflammatory effects^29,30^. However, the tentative identification of flavones like cirsimaritin (4), diosmetin (5), isorhamnetin (11) and hispidulin (13) suggests potential flavonoid contributions to *R. officinalis* bioactivity. Correlating the precise mass (m/z) of the pseudomolecular [M − H]^−^ and [M + H]^+^ ions for the peaks and their fragmentation patterns with previously published data in the literature was necessary to identify these compounds^31^.

**Fig. 2:**
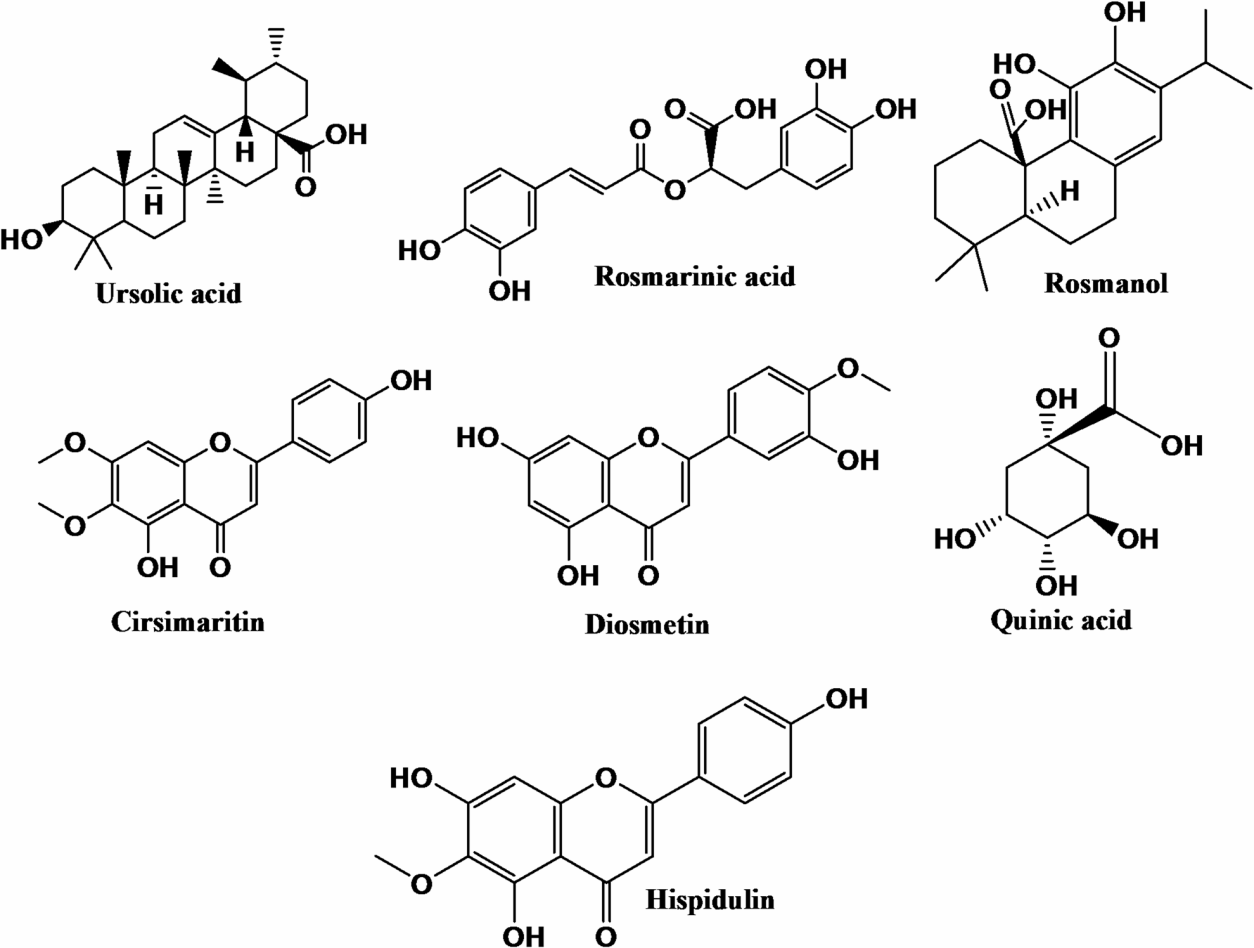
The chemical structures of the secondary metabolites identified in *R. officinalis* total extract.

**Fig. 3:**
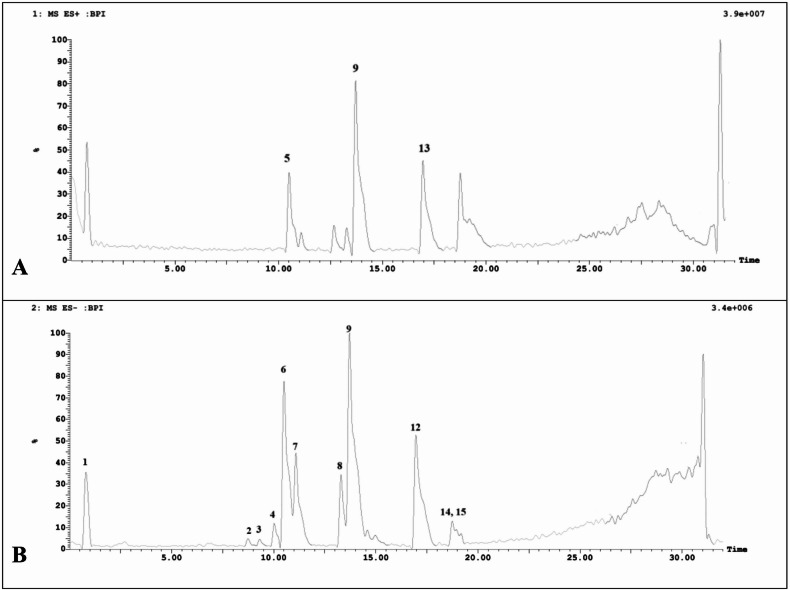
Total ion chromatogram of *R. officinalis* total methanol extract (**A**) in positive ion mode and (**B**) in negative mode.

Finally, the data underscores the *R. officinalis* rich phytochemical complexity, with compounds corroborated by literature, supporting its potential use in nutraceutical or pharmacological applications. Figure 2 illustrates the chemical structure of the major identified compounds; Fig. 3 shows the total ion chromatogram of *R. officinalis* methanol extract in positive and negative modes. The MS data of the discovered compounds, numbered in accordance with their sequence of elution, along with their retention times, m/z, and molecular formulae, are summarized in Table 1.

Rosmarinic acid, flavonoids, and diterpenoids are natural compounds that synergistically enhance wound healing through their strong antioxidant properties, which neutralize damaging free radicals; their anti-inflammatory effects, which mitigate tissue damage and excessive immune responses; and their collagen-promoting capabilities, which facilitate tissue regeneration and structural repair, rendering them significant as multi-target therapeutic agents in wound management^12,14,15,27^.

**Table 1:** Major metabolites assigned in *R. officinalis* aerial parts total extract via HPLC-ESI-MS/MS in positive and negative ion modes.

No.	*R*_t_	[M-H]^−^	[M+ H]^+^	m/z	Tentative Compound identification	Molecular Formula	Class	Relative Amount (%)	Reference
1	0.75	191.05	–	85, 127	Quinic acid	C_7_H_12_O_6_	Organic acid	3.45	^10,31^
2	8.74	327.26	–	299, 285	Carnosol quinone	C_20_H_24_O_4_	Diterpene	0.34	^29^
3	9.31	329.28	–	285	Carnosol	C_20_H_26_O_4_	Diterpene	0.29	^31^
4	10.02	313.15	–	255, 225	Cirsimaritin	C_17_H_14_O_6_	Flavonoid	1.09	^10,32^
5	10.50	-	301.16	211, 239, 258, 283, 286	3,5,7-Trihydroxy-4′- methoxyflavone (Diosmetin)	C_16_H_12_O_6_	Flavonoid	4.61	^33,34^
6	10.50	345.24	–	284	Rosmanol	C_20_H_26_O_5_	Diterpene	9.98	^10,31^
7	11.09	345.20	–	301, 286	12-Methoxy-carnosic acid (Methyl carnosate)	C_21_H_30_O_4_	Diterpene	5.91	^30,31^
8	13.28	343.21	−	299, 243	Rosmadial	C_20_H_24_O_5_	Diterpene	3.21	^29,31^
9	13.71	359.24	361.2157	150,169,314	Rosmarinic acid	C_18_H_16_O_8_	Phenolic acid	29.08	^10,34^
10	14.59	343.20	–	299, 243	Rosmadial isomer	C_20_H_24_O_5_	Diterpene	0.72	^29,31^
11	14.97	315.28	–	300, 301	Isorhamnetin	C_16_H_12_O_7_	Flavonoid	0.72	^31^
12	16.95	345.26	–	284	Epirosmanol (Rosmanol isomer)	C_20_H_26_O_5_	Diterpene	9.18	^10^
13	17.25	-	301.1968	299, 284, 256	Trihydroxymethoxyflavone (Hispidulin)	C_16_H_12_O_6_	Flavonoid	2.31	^32,35^
14	18.76	455.43	–	**–**	Ursolic acid	C_30_H_48_O_3_	Pentacyclic Triterpenoid	0.90	^10^
15	18.98	455.40	–	327, 317, 353, 409, 437	Betulinic acid	C_30_H_48_O_3_	Pentacyclic Triterpenoid	0.57	^29^

### Total phenolics and total flavonoids content of *R. officinalis* different fractions

The results showed that the total extract contained the highest phenolic content, equivalent to 106.56 µg gallic acid eq/mg sample, followed by the ethyl acetate extract at 97.38 µg gallic acid eq/mg, and then dichloromethane at 59.19 µg gallic acid eq/mg. The hexane, butanol, and water extracts contained the lowest content, equivalent to 49.86 µg gallic acid eq/mg, 28.61, and 20.76 µg gallic acid eq/mg, respectively. Moreover, the ethyl acetate fraction had the highest flavonoid concentration, which was equivalent to 43.7 ± 3.14 µg Rutin eq/mg (Table 2).

**Table 2:** Total phenolics and total flavonoids content of *R. officinalis* different fractions.

Sample	Total phenolics µg gallic acid eq/mg sample	Total flavonoids µg Rutin eq/mg sample
ROH	49.86 ± 2.10	34.88 ± 0.85
ROE	97.38 ± 4.90	43.7 ± 3.14
RON	28.61 ± 2.53	11.2 ± 0.49
ROT	106.56 ± 2.73	20.87 ± 1.22
ROD	59.19 ± 4.24	18.97 ± 0.99
ROA	20.76 ± 0.17	0.82 ± 0.07

Values are reported as Mean ± Standard error of three parallel measurements. ROH: *Rosmarinus officinalis* Hexane, ROE: *R. officinalis* Ethyl acetate, RON: *R. officinalis n*-butanol, ROT: *R. officinalis* total extract, ROD: *R. officinalis* dichloromethane, ROA: *R. officinalis* aqueous.

### *In vitro* antioxidant activity of *R. officinalis* different fractions

The antioxidant activity of the various fractions under study was measured through the FRAP assay, and it was found that the the antioxidant potential of *R. officinalis* varies significantly across different extraction fractions where the *n*-butanol fraction (RON) and the total extract (ROT) had the strongest antioxidant activity, with values ​​of 637.727 and 604.727 µM TE/ mg, respectively This was followed by the dichloromethane (ROD) and ethyl acetate (ROE), with values ​​of 542.832 and 422.57µM TE/ mg, respectively. In contrast, the ethyl acetate fraction (ROE) shows superior DPPH radical scavenging with an IC_50_ of 22.81 ± 1.01 *µg/*mL, while in the ABTS assay, the IC_50_ value equals 33.6 ± 1.08 *µg/*mL. The effectiveness of ethyl acetate extraction is attributed to its optimal polarity for recovering flavonoids and other phenolic antioxidants while excluding interfering compounds^36^. The ROD also had high antioxidant activity against both DPPH and ABTS, with IC_50_ values of 45.86 ± 1.04 and 32.36 ± 1.02 *µg/*mL, respectively as compared to Trolox with IC_50_ values of 5.248 ± 1.03 and 14.31 ± 1.02 *µg/*mL, respectively (Table 3).

These findings, supported by comprehensive antioxidant assays including FRAP, ABTS, and DPPH methods, reveal that solvent polarity and extraction methodology critically influence the recovery of bioactive compounds responsible for antioxidant activity. The research demonstrates that polar and semi-polar solvents, particularly ethyl acetate and *n*-butanol, are most effective at extracting flavonoids and phenolic compounds that contribute to the pronounced antioxidant properties of *R. officinalis*^37^. The comprehensive analysis of *R. officinalis* antioxidant potential across different extraction fractions reveals significant variation in bioactivity that correlates with solvent polarity and phytochemical composition. The *n*-butanol fraction demonstrates exceptional FRAP activity, while ethyl acetate fractions excel in DPPH radical scavenging and flavonoid content. These findings emphasise the importance of extraction method selection based on intended applications and target antioxidant mechanisms.

**Table 3:** *In vitro *antioxidant efficacy of different *R. officinalis* fractions.

Sample	DPPH assay IC_50_ µg/mL	ABTS assay IC_50_ µg/mL	FRAP assay µM TE/ mg
ROH	75.98 ± 1.05*	18.32 ± 1.03*	171.061 ± 5.25^a^
ROE	22.81 ± 1.01*	33.6 ± 1.08*^, a^	422.57 ± 18.92
RON	216.8 ± 1.03*	40.84 ± 1.03*	637.727 ± 64.28^b^
ROT	53.62 ± 1.04*	43.62 ± 1.04*	604.727 ± 50.90^b^
ROD	45.86 ± 1.04*	32.36 ± 1.02*^, a^	542.832 ± 16.48^b^
ROA	402.5 ± 1.03*	35.82 ± 1.02*^, a^	167.382 ± 14.02^a^
Trolox	14.31 ± 1.02	5.248 ± 1.03	-

Values are reported as Mean ± Standard error of three parallel measurements. TE: Trolox equivalent; ROH: *Rosmarinus officinalis* Hexane, ROE: *R. officinalis* Ethyl acetate, RON: *R. officinalis n*-butanol, ROT: *R. officinalis* total extract, ROD: *R. officinalis* dichloromethane, ROA: *R. officinalis* aqueous. Means bearing *are significantly different from Trolox (*p* < 0.05), Means bearing the same scripts (a), (b) are not significantly different from each other (*p* < 0.05).

### Anti-inflammatory effects of *R. officinalis* different fractions against LPS-induced NO in RAW 264.7 macrophages

#### Cytotoxicity assay

The SRB assay was used to assess the cytotoxic effects of the various *R. officinalis* fractions on RAW 264.7 murine macrophages. The cytotoxic effect was tested to establish the appropriate concentration ranges of RO different fractions for the analysis of ongoing anti-inflammatory potential; their cytotoxicity results on RAW 264.7 macrophages are represented in (Table 4). The fractions RON and ROA showed the highest viability with 84.56 ± 1.75% and 92.07 ± 1.08% viability at 100 *µ*g/ mL, respectively. While ROE and ROT showed the highest viability with 97.88 ± 1.12% and 97.31 ± 0.62% viability at 10 *µ*g/ mL, respectively.

It was found that neither fraction affected the viability of RAW 264.7 cells indicating that their inhibitory effects were not due to any cytotoxic effects.

**Table 4:** Cytotoxicity of *R. officinalis* different fractions against RAW 264.7 macrophages.

Sample	Viability%			
	100 µg/mL	10 µg/mL	1 µg/mL	0.1 µg/mL
ROH	2.69 ± 0.30^a^	93.10 ± 1.08^a^	94.14 ± 2.80^a^	95.14 ± 0.83^a^
ROE	3.60 ± 0.3^a, b^	97.88 ± 1.12^b^	99.21 ± 2.38^b^	100.71 ± 0.62^b^
RON	84.56 ± 1.75	87.42 ± 1.55^c^	91.63 ± 1.81^a, c^	92.50 ± 1.41^a^
ROT	19.44 ± 0.20	97.31 ± 0.62^b^	97.68 ± 0.40^a, b^	98.57 ± 1.23^b^
ROD	3.87 ± 0.06^b^	92.69 ± 1.25^a^	93.55 ± 1.40^a^	93.71 ± 1.22^a^
ROA	92.07 ± 1.08	88.22 ± 0.86^c^	88.51 ± 1.17^c^	88.43 ± 1.67

Values are reported as Mean ± Standard error of three parallel measurements. ROH: *Rosmarinus officinalis* Hexane, ROE: *R. officinalis* Ethyl acetate, RON: *R. officinalis n*-butanol, ROT: *R. officinalis* total extract, ROD: *R. officinalis* dichloromethane, ROA: *R. officinalis* aqueous. Means bearing the same scripts are not significantly different from each other (*p* < 0.05). Comparison is done according to concentrations.

#### Nitric oxide Inhibition assay

To study the effect of *R. officinalis* different fractions, the Griess assay was employed to quantify nitrite formation in the culture medium. All studied fractions of *R. officinalis* inhibited LPS-induced NO generation in macrophage cells in a concentration-dependent manner (Table 5). The highest inhibitory activity has been recorded in ROE and ROD with 70.88 ± 1.63% and 72.48 ± 3.92%, respectively, at 10 *µg/*mL. Followed by ROH with 64.89 ± 2.52% at the same concentration as compared to positive control L-NG-nitro arginine methyl ester (L-NAME) (1 mM) that demonstrated 84.64 ± 1.04% NO inhibition.

The anti-inflammatory potential of *R. officinalis* fractions demonstrates significant variation depending on solvent polarity and phytochemical composition, with distinct mechanisms of action observed across extraction phases. Phenolic diterpenes (carnosic acid, carnosol) and rosmarinic acid are primary contributors to *R. officinalis* anti-inflammatory activity, Suppression of nuclear translocation reduces expression of pro-inflammatory enzymes (COX-2, iNOS) and cytokines (TNF-α, IL-6) ^38,39^. Also, carnosic acid showed inhibition of iNOS by 72% at 6.2 *µ*g/mL and complete inhibition at > 12.5 *µ*g/mL^40^.

**Table 5:** Anti-inflammatory effects of *R. officinalis* different fractions against nitric oxide.

Sample	Inhibition of NO % (Mean ± S.E)				IC_50_ µg/mL
	100 µg/mL	10 µg/mL	1 µg/mL	0.1 µg/mL	
ROH	–	64.89 ± 2.52*^, a^	9.64 ± 0.62*^, a^	9.25 ± 0.62*^, a^	5.96
ROE	–	70.88 ± 1.63*^, a^	14.53 ± 2.49*^, a^	6.18 ± 0.77*^, a^	4.56^a^
RON	15.74 ± 2.43^a^	13.74 ± 2.06*	9.81 ± 3.15*	8.52 ± 3.07*	–
ROT	–	32.36 ± 3.77*	5.57 ± 0.94*	3.81 ± 2.43*	–
ROD	–	72.48 ± 3.92*^, a^	13.71 ± 1.63*^, a^	3.42 ± 0.43*	4.48^a^
ROA	16.91 ± 1.28^a^	15.99 ± 2.34*	13.95 ± 0.95*	13.40 ± 1.45*	–
L-NAME (1 mM)	84.64 ± 1.04				–

Values are reported as Mean ± Standard error of three parallel measurements. ROH: *Rosmarinus officinalis* Hexane, ROE: *R. officinalis* Ethyl acetate, RON: *R. officinalis n*-butanol, ROT: *R. officinalis* total extract, ROD: *R. officinalis* dichloromethane, ROA: *R. officinalis* aqueous. Means bearing *p < 0.05 are significantly different from the standard NAME. Means bearing the same scripts (^a^) are not significantly different from each other at *p* < 0.05. Comparison is done according to concentrations. (−) means unspecified IC_50_ *µ*g/ mL.

### Wound healing activity of *R. officinalis* different fractions

#### Cytotoxicity assay

**Fig. 4:**
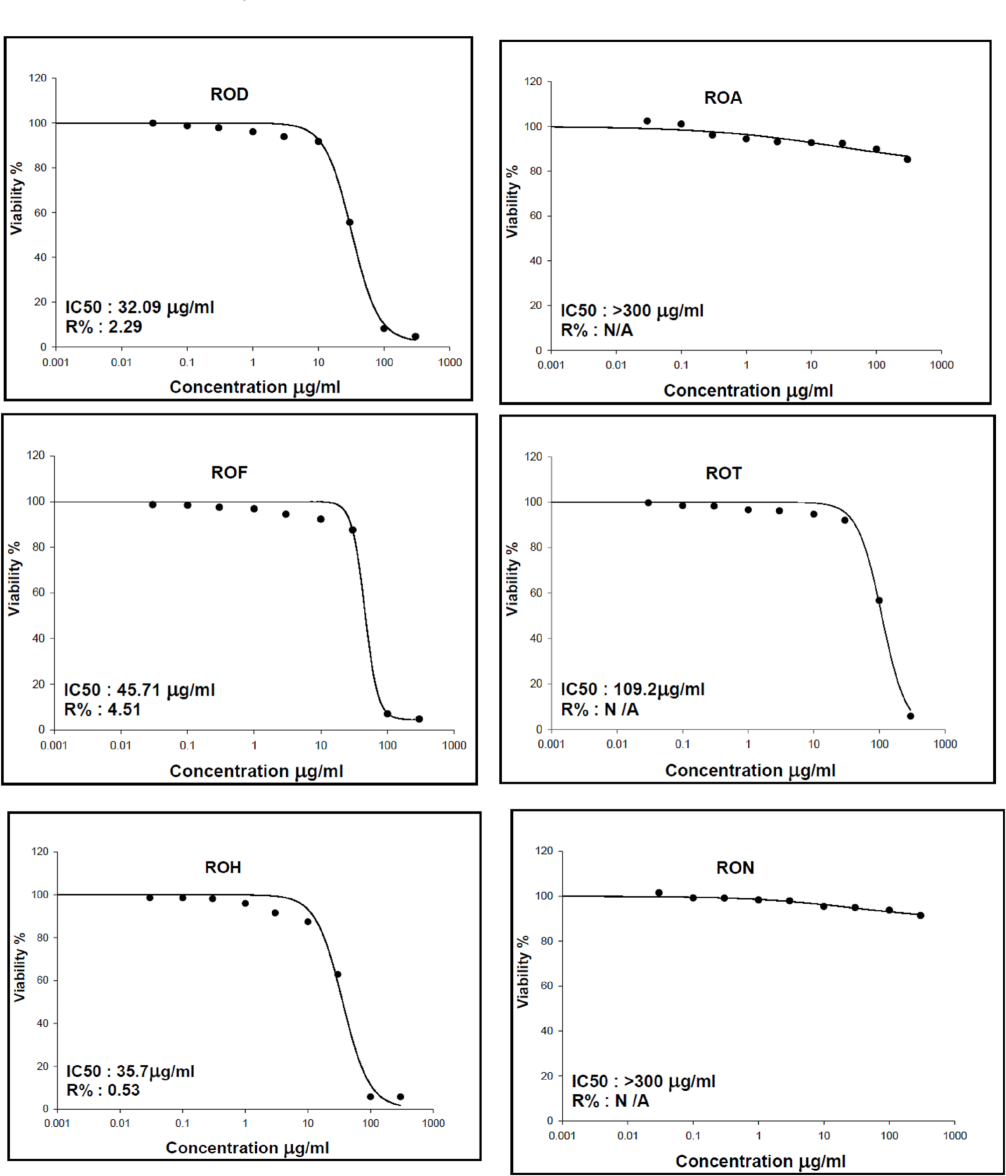
The effect of *R. officinalis* different fractions on Human Skin Fibroblast cells (HSF) viability. ROH: *Rosmarinus officinalis* Hexane, ROE: *R. officinalis* Ethyl acetate, RON: *R. officinalis n*-butanol, ROT: *R. officinalis* total extract, ROD: *R. officinalis* dichloromethane, ROA: *R. officinalis* aqueous.

Assessing the cytotoxicity of various fractions of R. officinalis on HSF cells is essential to evaluate the safety of the treatment dosage. The cytotoxic effects of various fractions on HSF cells were assessed utilizing the MTT test^41^. In our study, ROA and RON (up to 100 *µ*g/mL) shown no cytotoxicity towards HSF cells. All evaluated fractions exhibited cell viability exceeding 90% at a concentration of 10 *µg/*mL, with the exception of ROH, which demonstrated a viability of 87.33% at the same dose (Fig. 4). The concentration of 10 *µ*g/mL was deemed safe and subsequently utilized as the treatment dose in the scratch wound assay.

#### Scratch wound assay

**Fig. 5:**
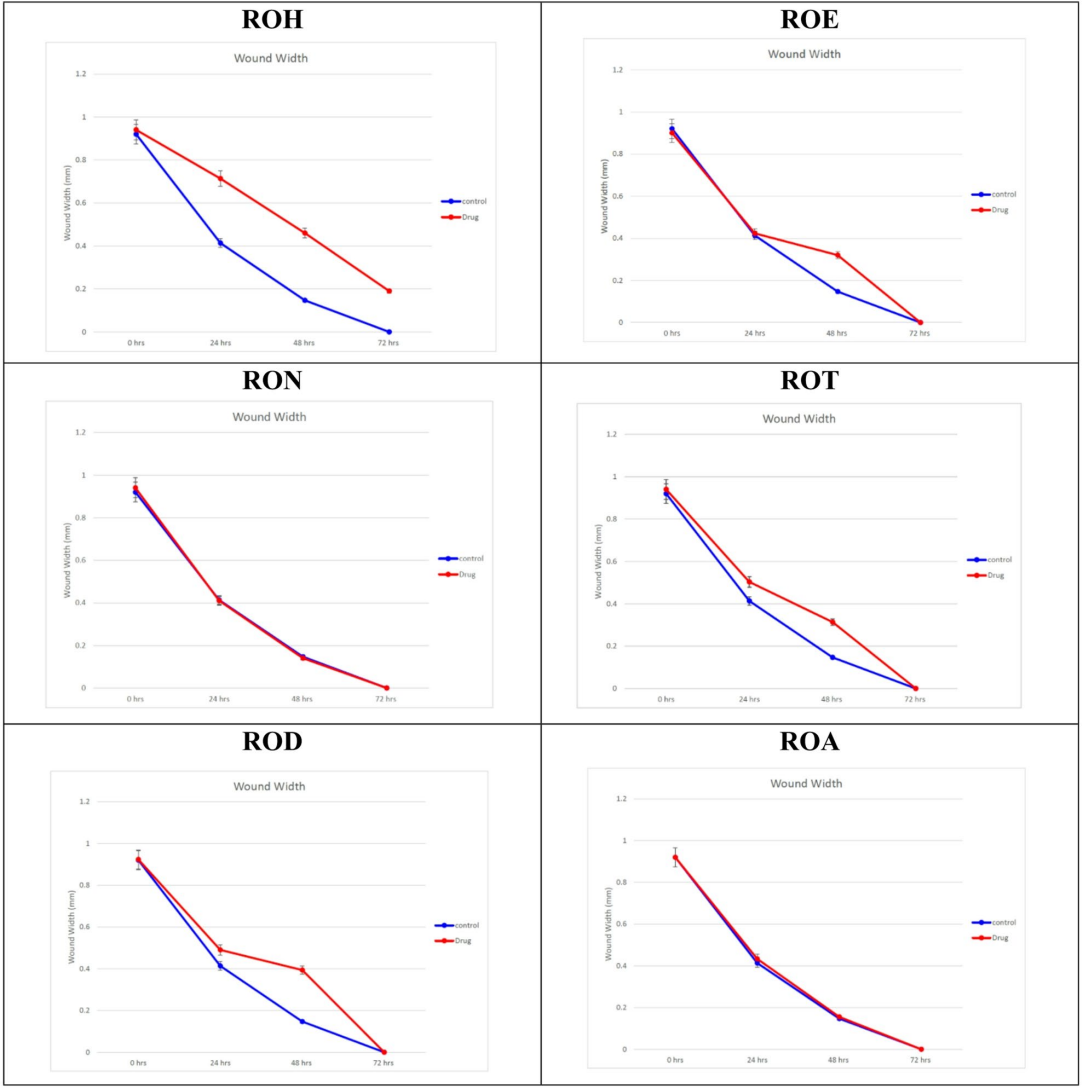
The wound width changes in the absence or presence of *R. officinalis*, different fractions (10 *µ*g/mL). ROH: *Rosmarinus officinalis* Hexane, ROE: *R. officinalis* Ethyl acetate, RON: *R. officinalis n*-butanol, ROT: *R. officinalis* total extract, ROD: *R. officinalis* dichloromethane, ROA: *R. officinalis* aqueous.

**Fig. 6:**
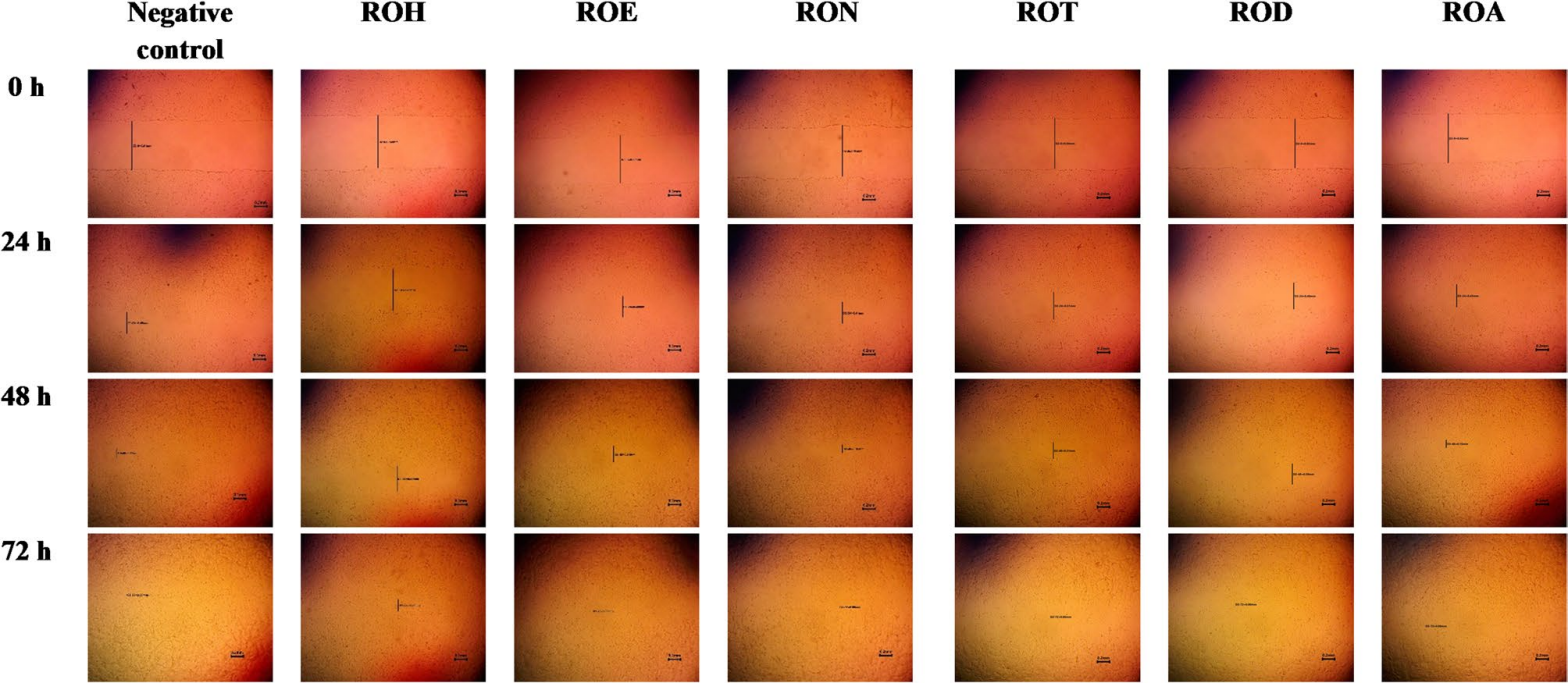
Microscopical representative images for wound healing of *R. officinalis*, different fractions (10 *µ*g/mL). ROH: *Rosmarinus officinalis* Hexane, ROE: *R. officinalis* Ethyl acetate, RON: *R. officinalis n*-butanol, ROT: *R. officinalis* total extract, ROD: *R. officinalis* dichloromethane, ROA: *R. officinalis* aqueous.

This work employed an *in vitro* scratch assay with HSF cells to evaluate the wound-healing efficacy of various fractions of R. officinalis. The assessment was conducted by measuring the average distance between the edges of the wounds to determine changes in width (Fig. 5). The evaluated fractions at a concentration of 10 *µ*g/mL significantly reduced the wound width compared to the control cells (Table 6; Fig. 6). After 24 h, the highest wound healing potential was recorded by ROE, RON and ROA with wound widths of 0.43 ± 0.005, 0.41 ± 0.005 and 0.42 ± 0.04 mm, respectively. Subsequently, ROD and ROT exhibited wound widths of 0.49 ± 0.005 mm and 0.50 ± 0.01 mm, respectively, in contrast to the control cells, which had a wound width of 0.41 ± 0.02 mm. As the wound width decreased with greater cell migration, our findings indicated that both RON and ROA demonstrated effective cell migration among the tested fractions, with wound widths of 0.14 and 0.15 mm, respectively, after 48 h. All examined fractions demonstrated nearly complete cell migration after 72 h of observation, with the exception of ROH. This indicated that an increased polar percentage correlates with improved wound healing effects, seen by enhanced fibroblast migration.

Rosmarinic acid can be attributed to the observed wound healing potential of different fractions, where previous studies in an animal model revealed its anti-inflammatory action, which reduces leukocyte infiltration and tissue degeneration, thereby supporting a more favourable environment for tissue repair, improved healing, as evidenced by reduced inflammation, edema, and vascular dilatation^42,43^.

**Table 6:** Wound width of scratched human skin fibroblast (HSF) cells treated without *R. officinalis* extracts (negative control) and with various *R. officinalis* fractions (10 *µ*g/mL).

Time (h)	Wound width (mm)						
	ROH	ROE	RON	ROT	ROD	ROA	Control
	10 µg/mL						
0	0.94 ± 0.005	0.90 ± 0.005	0.94 ± 0.1	0.94 ± 0.1	0.92 ± 0.005	0.92 ± 0.01	0.92 ± 0.01
24	0.71 ± 0.05^a^	0.42 ± 0.04	0.41 ± 0.005	0.50 ± 0.01^a^	0.49 ± 0.005^a^	0.43 ± 0.005	0.41 ± 0.02
48	0.46 ± 0.07^b^	0.32 ± 0.01^b^	0.14 ± 0.01	0.31 ± 0^b^	0.39 ± 0.005^b^	0.15 ± 0.007	0.14 ± 0.02
72	0.19 ± 0.07^c^	0^c^	0^c^	0^c^	0^c^	0^c^	0.08 ± 0.02

Values are reported as Mean ± Standard error of three parallel measurements. ROH: *Rosmarinus officinalis* Hexane, ROE: *R. officinalis* Ethyl acetate, RON: *R. officinalis n*-butanol, ROT: *R. officinalis* total extract, ROD: *R. officinalis* dichloromethane, ROA: *R. officinalis* aqueous. (^a^) significant difference from the control group in the time interval (24 h). (^b^) significant difference from the control group of the time interval (48 h). (^c^) significant difference from the control group of the time interval (72 h).

### Network pharmacology-based analysis of *R. officinalis* extract in wound healing

#### Screening of Rosmarinic acid and wound healing-related target genes

**Fig. 7:**
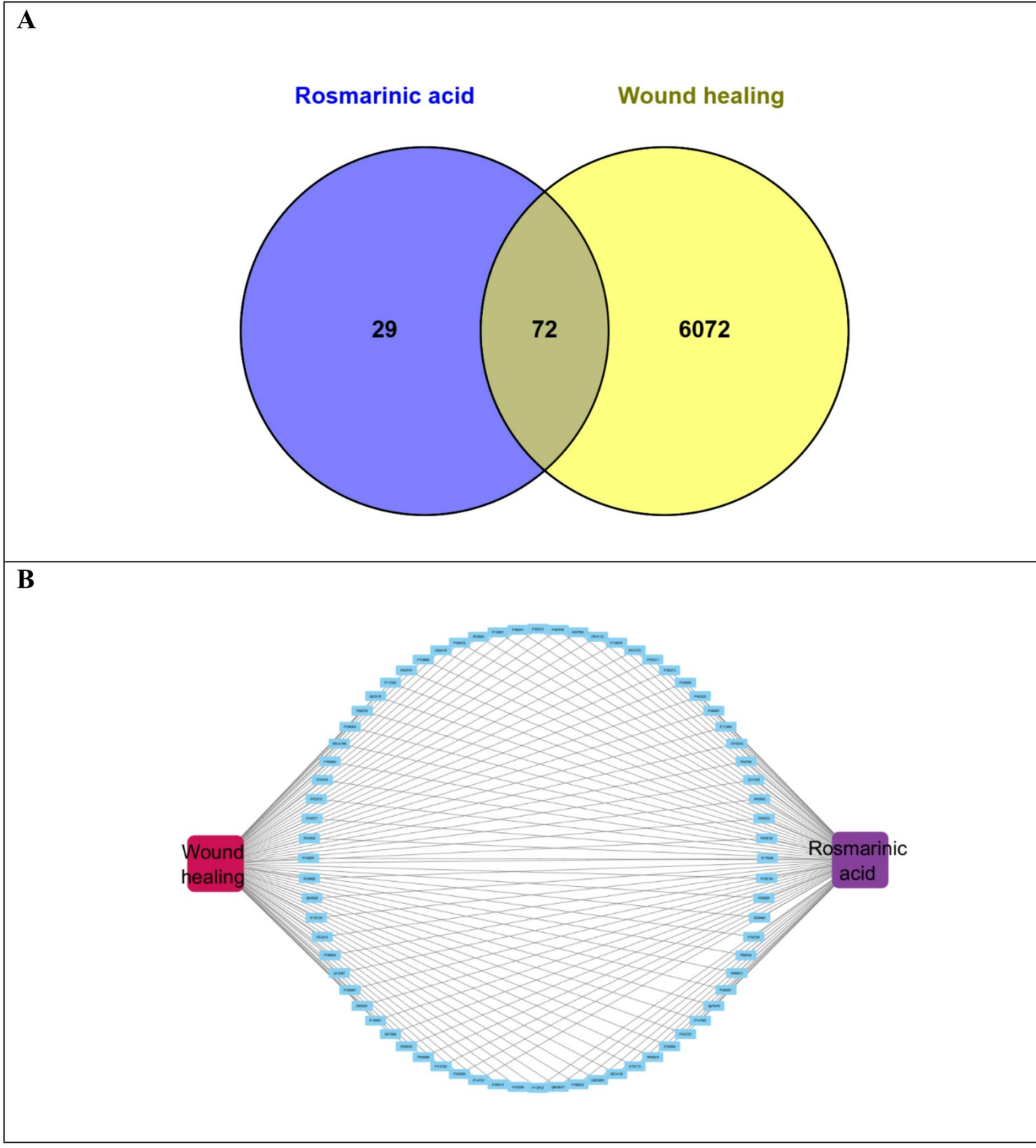
Venn diagram for the integrated analysis of the related targets of rosmarinic acid and wound healing **(A)**; The rosmarinic acid-target-wound healing network **(B)**.

Predicted 101 target genes related to rosmarinic acid were collected from Swiss Target Prediction and these genes were converted into their conical gene names using the UniProt database. A total of 6144 common target genes known to play a role in wound healing were gathered from GeneCards databases using the keywords “wound healing” and the species limited to “Homo sapiens”. Duplicate targets were removed, and the Venn diagram was created to compare the targets regulated by rosmarinic acid and the potential targets for wound healing, it revealed a total of 72 common intersection targets in (Fig. 7A).

The drug-targets and disease-targets lists were imported into Cytoscape to draw a compound–target-disease network diagram (Fig. 7B). This network is composed of 74 nodes linked via 142 edges, which revealed the synergistic multicomponent and multitargeted effects of rosmarinic acid contributing to its wound healing activity (Table 7).

**Table 7:** The core compound, Rosmarinic acid, and the top 10 core targets in the management of wound healing.

Name	Degree	Betweenness Centrality	Closeness Centrality
Rosmarinic acid	70	0.459474886	0.935897
P13612	2	1.63E-05	0.506944
P09960	2	1.63E-05	0.506944
P35354	2	1.63E-05	0.506944
P42892	2	1.63E-05	0.506944
P30556	2	1.63E-05	0.506944
P08709	2	1.63E-05	0.506944
P10828	2	1.63E-05	0.506944
Q9ULW8	2	1.63E-05	0.506944
P10827	2	1.63E-05	0.506944
Q13547	2	1.63E-05	0.506944

#### Protein–protein interaction (PPI) network construction

**Fig. 8:**
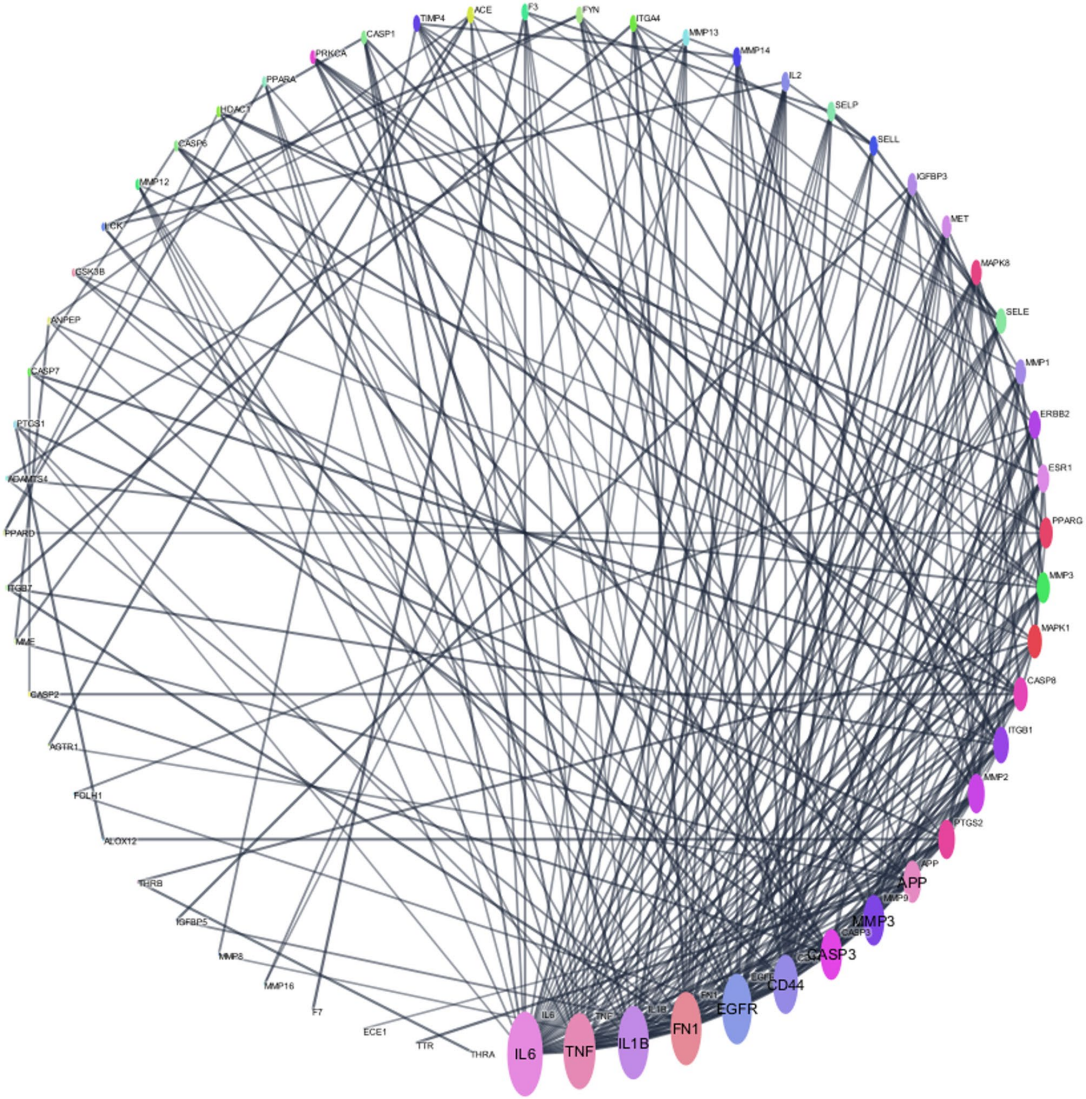
Network nodes represent 60 protein targets, and the edges represent protein–protein interactions. The size of nodes signifies the connectivity of each protein, the higher the node size the higher its connectivity to other nodes.

**Fig. 9:**
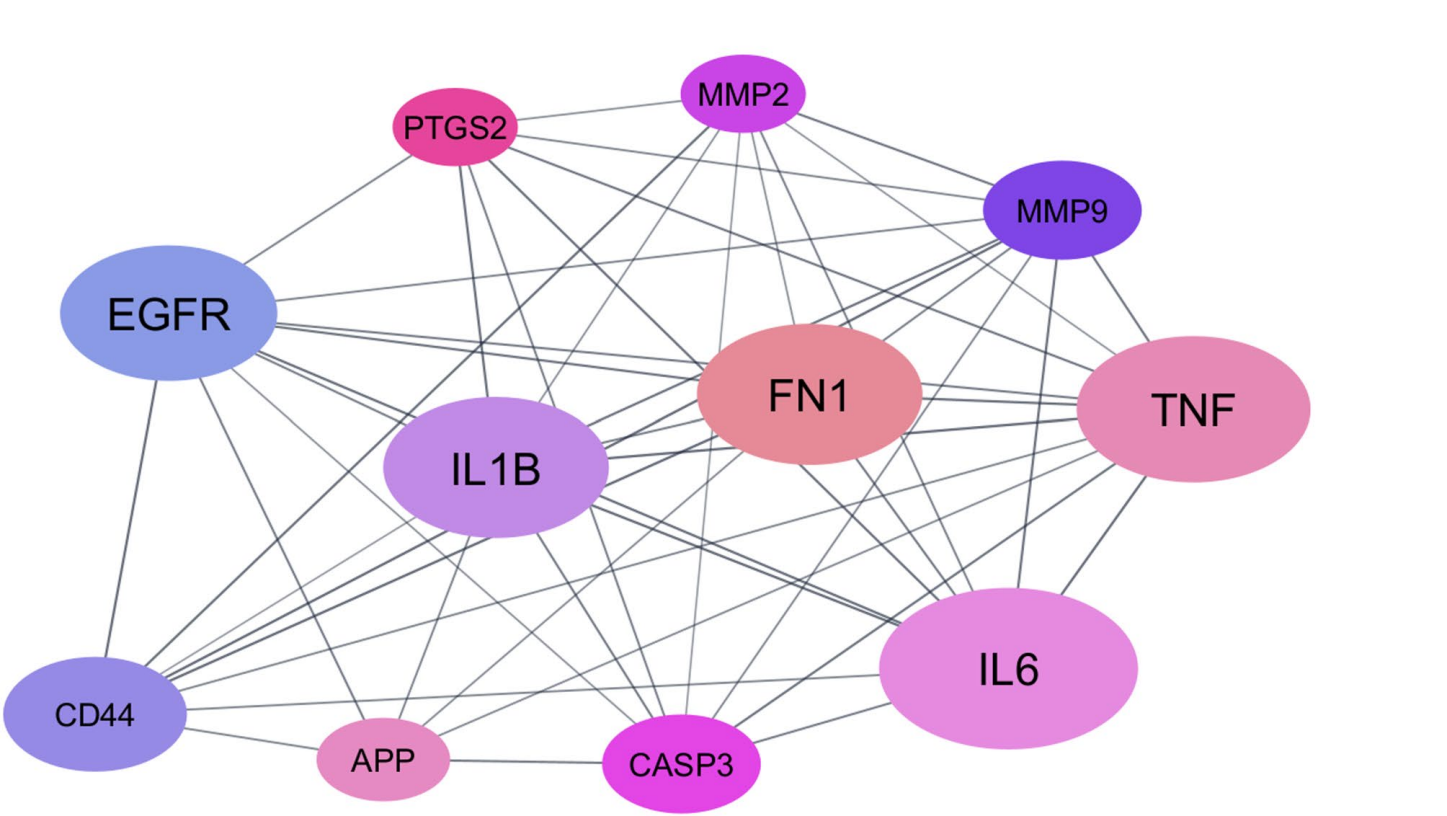
PPI subnetwork after network analysis and filtration (Nodes whose degree value > 15 are exposed). The node size is related to the degree.

The 72 overlapping target genes were imported into the STRING database to perform an analysis of protein–protein interactions (PPI). The results were used to generate a PPI network diagram using the Cytoscape 3.10.0 software, resulting in 60 nodes (after excluding nodes that were not connected), 269 edges, and the average node connectivity was found to be 8.967 as depicted in (Fig. 8). Afterwards, a core PPI subnetwork composed of 11 genes with the highest degree values was constructed as depicted in (Fig. 9), namely IL6, TNF, FN1, IL1B, EGFR, CD44, CASP3, MMP9, APP, PTGS2 and MMP2. We summarised the topological parameters such as node degree, betweenness, and closeness for each protein as well as skin tissue expression score in (Table 8).

Effective wound healing depends on IL-6 because it strictly regulates inflammation, cellular proliferation, collagen synthesis, and the development of new blood vessels^44,45^. Also, TNF-α is essential for starting inflammation, promoting tissue regeneration, and regulating cellular responses required for efficient wound healing^46,47^. IL-1β is crucial in wound healing since it facilitates the initial inflammatory response essential for pathogen eradication and tissue regeneration. It promotes the induction of neutrophils and macrophages, stimulates the proliferation of fibroblasts and keratinocytes, and regulates extracellular matrix remodeling by influencing collagen and other matrix proteins. IL-1β additionally facilitates angiogenesis by stimulating the production of vascular endothelial growth factor (VEGF)^48,49^. Fibronectin 1 helps produce extracellular matrix (ECM) and assist cell adhesion, migration, and proliferation during wound healing. It is part of the provisional fibrin-fibronectin matrix that forms early in wound healing, allowing fibroblasts and endothelial cells to migrate into the wound and promote tissue regeneration and angiogenesis. Fibernectin activates macrophages to remove debris and helps epithelial cells migrate for re-epithelialization. Fibronectin is replaced by mature ECM components like collagen during remodeling, strengthening the scar and restoring tissue integrity. Its diverse role is crucial for wound healing and tissue regeneration^50^.

**Table 8:** The hub targets of Rosmarinic acid in wound healing management and the topological parameters.

Target	Name	Degree	Betweenness Centrality	Closeness Centrality
IL6	Interleukin‑6	31	0.116608	0.655555556
TNF	Tumor Necrosis Factor	28	0.102569	0.648351648
FN1	Fibronectin 1	27	0.129476	0.614583333
IL1B	Interleukin‑1B	27	0.107507	0.627659574
EGFR	Epidermal Growth Factor Receptor	26	0.110692	0.614583333
CD44	Cell-surface glycoprotein	22	0.057791	0.567307692
CASP3	Caspase-3	19	0.077799	0.556603774
MMP9	Matrix metalloproteinase‑9	19	0.0279	0.561904762
APP	Amyloid‑beta A4 protein	16	0.136419	0.551401869
PTGS2	Prostaglandin epoxide synthase	15	0.041969	0.51754386
MMP2	Matrix metalloproteinase‑2	15	0.013241	0.526785714

## Materials and methods

### Plant material and Preparation of different extracts

The aerial parts of *R. officinalis* have been collected in October 2024 from the medicinal plants experimental site at the Faculty of Pharmacy, Cairo University. Samples were thankfully recognized and authenticated by Eng. Terase Labib, an agricultural engineer who works as a consultant for plant taxonomy at the Ministry of Agriculture and the El-Orman Botanical Garden in Giza, Egypt. The voucher specimen (BUC-PHG-RO-23) was preserved at the Department of Pharmacognosy, Badr University, Cairo, Egypt.

The plant material (150 g) was macerated in 80% methanol. After the extraction procedures, the extract was filtered utilizing Whatman No.1 filter paper. Using a rotary evaporator, the solvent was removed, yielding (ROT) 18.993 g ^51^. The total extract was subjected to solvent-solvent fractionation starting with the defatting step using *n*-hexane (ROH), followed by dichloromethane (ROD), ethyl acetate (ROE), and *n*-butanol (RON) and the remaining is the aqueous fraction (ROA). All fractions were stored at 4 °C until analysis.

### HPLC-ESI-MS/MS analysis

The metabolic profiles of *R. officinalis* were examined using high-performance liquid chromatography and electrospray ionisation mass spectrometry on the whole extract. Analysis was done on Shimadzu^®^ 8045 HPLC-ESI-MS/MS with a C18 reversed phase column (Shimpack UPLC—2.7 μm, 2 × 150 mm). Shimadzu^®^ triple quadrupole mass analyzers acquired negative and positive ions. In HPLC-grade methanol, samples were dissolved and filtered over a 0.2 μm PTFE membrane. The following is how MS-grade mobile phases were used: A: 0.1% formic acid (v/v) in water, and B: 0.1% formic acid (v/v) in methanol. With a flow rate of 0.2 mL/min, the elution profile was as follows: 0–2 min, 10% B (isocratic); 2–5 min, 10–30% B in A; 5–15 min, 30–70% B in A; 15–22, 70–80% B in A; 22–26, 80% B in A (isocratic); 29–30 min, 80–10% B in A; and 30–35 min, 10% B in A (isocratic). Mass detection was done from 100 to 1200 m/z. Set the ion source temperature to 200 °C, capillary voltage to 3000 eV, desolvation and interface temperatures to 526 and 300 °C, respectively. Cone gas flow was 50 L/h, nebulizing gas 3 L/min. MS/MS measurements were taken for collision-induced dissociation (CID). For each mass peak, the fragmentation cone voltage was changed from 10 to 40 eV. Data were processed with LabSolutions^52–55^.

### Total phenolics and total flavonoids content

The Folin–Ciocalteu technique was used to calculate the total phenolic content. Briefly, the procedure consisted of mixing 10 µL of sample/standard with 100 µL of Folin-Ciocalteu reagent (Diluted 1: 10) in a 96-well microplate. Then, 80 µL of 1 M Na_2_CO_3_ was added and incubated at room temperature (25 °C) for 20 min in the dark. At the end of incubation time, the resulting blue complex color was measured at 630 nm. Data are represented as means ± SD. The results were recorded using a microplate reader FluoStar Omega^56^.

With a few adjustments, the aluminum chloride method was used to determine the total flavonoid concentration in microplates. In short, a 96-well microplate was filled with 15 µL of sample/standard, 175 µL of methanol, and 30 µL of 1.25% AlCl_3_. After that, 30 µL of 0.125 MC_2_H_3_NaO_2_ was added, and the mixture was incubated for five minutes. At 420 nm, the resultant yellow color was detected after the incubation period. Means ± SD are used to represent data.A FluoStar Omega microplate reader was used to capture the results^57^.

### Antioxidant potential using FRAP assay, DPPH, ABTS assay

The sample’s ability to convert ferric ions to ferrous ions is a measure of its potential antioxidant activity, or ferric reducing antioxidant power (FRAP). The previously described methodology was used to conduct the assay^58,59^, with modest microplate adjustments. A freshly made TPTZ reagent was used: 300 mM acetate buffer (pH 3.6), 10 mM TPTZ in 40 mM HCl, and 20 mM FeCl3, in a volumetric ratio of 10:1:1. In a 96-well plate (*n* = 3), 190 µL of newly made TPTZ reagent was mixed with 10 µL of sample. The reaction was incubated at 30 min at ambient temperature without light. The blue color was measured at 593 nm after incubation. Data are shown as means ± SD ^58,59^.

The ABTS assay was carried out adopting the modifications of Elkholy et al. ^58^ to be carried out in microplates, briefly; 192 mg of ABTS were dissolved in distilled water and transferred to a 50 mL volumetric flask, which was filled with distilled water. Mix 1mL of the previous solution with 17 µL of 140 mM potassium persulphate and let it sit for 24 h in the dark. The final ABTS dilution for the assay was 1mL of the reaction mixture diluted to 50 mL with methanol. Mix 190 µL of newly produced ABTS reagent with 10 µL of sample/compound on a 96-well plate (*n* = 6) and incubate at room temperature. In darkness for 30 min. At 734 nm, ABTS color intensity decreased after incubation. Data are shown as means ± SD using the equation: % inhibition= ((Average blank-average test absorbance)/(Average blank)) *100 ^58,59^.

DPPH (2,2-diphenyl-1-picryl-hydrazyl-hydrate) free radical assay was performed using the method of Boly et al. ^60^ adopting the modifications of Elkholy et al. ^58^. In summary, 100µL of newly made DPPH reagent (0.1% in methanol) was added to 100 µL of sample on a 96-well plate (*n* = 3) and incubated at room temperature for 30 min in the dark. The DPPH color intensity reduction at 540 nm after incubation was measured. Data are shown as means ± SD ^58,60^.

### *In vitro* Anti-inflammatory assay

#### Cytotoxicity assay

We obtained mouse RAW264.7 macrophage cells from Nawah Scientific Inc. in Mokattam, Cairo, Egypt.At 37 °C in a humidified environment with 5% (v/v) CO2, cells were grown in DMEM media with 100 mg/mL streptomycin, 100 units/mL penicillin, and 10% heat-inactivated fetal bovine serum. Cell viability was evaluated using the SRB assay. Aliquots of 100 µL cell suspension (5 × 10^3 cells) were placed in 96-well plates and incubated in complete media for 24 h.Cells were treated with an additional aliquot of 100 µL of media containing drugs at varying concentrations. To fix cells after 72 h of drug exposure, 150 µL of 10% TCA was added to the medium and incubated at 4 °C for 1 h.The TCA solution was removed, and the cells were washed five times with distilled water. Add 70 µL SRB solution (0.4% w/v) and incubate at room temperature for 10 min in a dark atmosphere. Plates were air-dried overnight after three 1% acetic acid washes. After adding 150 µL of TRIS (10 mM) to solubilize protein-bound SRB stain, absorbance was measured at 540 nm using a BMGLABTECH^®^- FLUOstar Omega microplate reader (Ortenberg, Germany)^61^.

#### Nitric oxide inhibitory assay

RAW264.7 cells were incubated in 96-well plates for 24 h. Inflammation was produced with 1 *µ*g/mL LPS the next day, while untreated cells were renewed with fresh medium (Control group). LPS will be applied at 2–5 concentrations to compounds. Equal quantities of cell supernatant and Griess reagent were incubated in the dark at room temperature for 10 min to assess NO secretion. L-NAME (L-NG-nitro arginine methyl ester) (1 mM) was used as a standard NOS inhibitor. The ELISA plate reader measured nitric content by measuring absorbance at 540 nm^62^.

### Wound healing activity

#### Cytotoxicity assay

The 3-(4, 5-dimethylthiazolyl-2)-2, 5-diphenyltetrazolium bromide (MTT) assay was used to investigate the cytotoxicity of *R. officinalis* fractions against the HSF (Human Skin Fibroblast) cell line before the wound-healing assay. The HSF cell line came from Nawah Scientific Inc. in Mokatam, Cairo, Egypt. We cultivated cells in DMEM media with 100 mg/ml streptomycin, 100 units/ml penicillin, and 10% heat-inactivated fetal bovine serum in a humidified 37 °C atmosphere with 5% (v/v) CO2. For 24 h, 100 µL cell suspension (5 × 103 cells) was incubated in 96-well culture plates at 37 °C with 5% CO_2_. The cells were treated with a 100 µL aliquot of media containing various concentrations of tested materials (0.01, 0.1, 1, 10, and 100 *µ*g/mL). Remove medium and add 20 µl of 1 mg/ml MTT solution to PBS in each well after 48 h. At 37 °C, incubate 4 h. After producing formazan crystals, dilute them in 100 µL pure DMSO. Measured formazan solution absorbance at λmax 570 nm using a BMGLABTECH^®^-FLUOstar Omega microplate reader (Omega, Germany)^63^.

#### Scratch wound assay

*In vitro* HSF cell migration experiments assessed the wound-healing efficacy of *R. officinalis* fractions. For scratch wound assay, cells were plated at 2 × 105/well on a coated 12-well plate and cultivated overnight in 5% FBS-DMEM at 37 °C and 5% CO_2_. The next day, horizontal scratches were made in the confluent monolayer, the plate was rinsed with PBS, control wells were refilled with medium, and drug wells were refilled with drug-containing media. Inverted microscope images were taken at the stated times. Between time periods, the plate was incubated at 37 °C and 5% CO2. The experiment was tripled. The average distance between scratch edges determines wound width, which decreases with cell migration. These results are shown as mean ± SD ^64–66^.

### Network pharmacology-based analysis of *R. officinalis* extract in wound healing

#### Screening of Rosmarinic acid-related target genes

Target genes for rosmarinic acid were determined using the respective Simplified Molecular Input Line Entry Specification (SMILES). The targets of rosmarinic acid were predicted using Swiss Target Prediction (http://www.swisstargetprediction.ch/ )^67^, and SEA (Similarity Ensemble Approach) (https://sea.bkslab.org/ ).

#### Screening of wound healing process‑related target genes

Genes associated with the wound healing process were collected from the GeneCards database GeneCards (https://www.genecards.org/ (^68^ and UniProt (https://www.uniprot.org/) ^69^using the keyword " wound healing” and the duplicates were deleted. Venn diagrams (https://bioinfogp.cnb.csic.es/tools/venny/) were accessed in June 2025 and used to determine the intersection targets between compounds and disease. The compound-target-disease network was constructed using Cytoscape software.

#### Protein–protein interaction (PPI) network construction

Following the intersection, targets were screened, and their PPI network diagram was established using STRING (https://string-db.org/) ^70^. The PPI network was visualised using Cytoscape software (version 3.7.2) ^71^. With STRING scores, PPIs were categorised as high, medium, or low confidence. Confidence is the likelihood of protein interaction. We assigned class labels using probability levels of 0.8 to determine the confidence level of our PPI predictions^72^.

### Statistical analysis

All experiments were tripled. All data were presented as mean ± SEM. Results are analyzed using one-way ANOVA and Tukey’s post hoc test. All statistical analyses were done with GraphPad Prism 6.01 (San Diego, CA, USA). Statistical significance was defined as probability values ≤ 0.05.

## Conclusions

The wound healing efficacy of *R. officinalis* fractions is inextricably linked to solvent-specific phytochemical profiles and antioxidant mechanisms. Ethyl acetate (ROE) and *n*-butanol (RON) fractions emerged as superior candidates, driven by high rosmarinic acid, diterpenoid, and flavonoid content. ROE’s dual role in radical scavenging (DPPH/ABTS) and fibroblast migration acceleration (53.3% wound closure at 24 h) positions it as a lead fraction for acute wound repair. Concurrently, RON’s exceptional FRAP activity (637.727 µM TE/mg) highlights its utility in redox-mediated tissue regeneration. The anti-inflammatory synergy of these fractions, evidenced by > 70% NO inhibition in macrophages, further supports their mechanistic role in mitigating chronic inflammation. Critically, the study underscores the limitations of total extracts (ROT), which, despite high phenolic content, underperformed compared to optimized fractions. A network of 72 genes associated with wound healing was established through protein–protein interactions (PPI). Among these genes, IL6, TNF, FN1, IL1B, EGFR, CD44, CASP3, MMP9, APP, PTGS2 and MMP2 demonstrated significance in terms of their connectivity within the network. Future research should prioritise in vivo validation of fraction-specific formulations and clinical translation, particularly for diabetic or infected wounds. Also, further investigation will be carried out to fully comprehend the mechanism of rosmarinic acid in wound healing such as elucidating how rosmarinic acid modulates signaling pathways involved in inflammation resolution as NF-κB and Nrf2, also in tissue regeneration targeting TGF-β and PI3K/Akt. Furthermore, investigating rosmarinic acid’s impact on extracellular matrix components like collagen and fibronectin, including effects on matrix metalloproteinases that regulate tissue remodeling. Standardising extraction protocols for ROE and RON could unlock *R. officinalis* full potential as a multifunctional natural therapeutic agent.

## Data Availability

This document includes all of the data on the measured ecosystem variables that show ecosystem functions and support the study’s conclusions. Requests for data can be made to the corresponding author (Aly, S.H.).
